# Development, implementation, and evaluation of a multi-addiction prevention program for primary school students in Hong Kong: the B.E.S.T. Teen Program

**DOI:** 10.1186/s40405-016-0014-z

**Published:** 2016-07-08

**Authors:** Daniel T. L. Shek, Lu Yu, Hildie Leung, Florence K. Y. Wu, Moon Y. M. Law

**Affiliations:** 1 Department of Applied Social Sciences, The Hong Kong Polytechnic University, Hunghom, Hong Kong, People’s Republic of China; 2 Centre for Innovative Programmes for Adolescents and Families, The Hong Kong Polytechnic University, Hong Kong, People’s Republic of China; 3 Department of Social Work, East China Normal University, Shanghai, People’s Republic of China; 4 Kiang Wu Nursing College of Macau, Macao, People’s Republic of China; 5 University of Kentucky College of Medicine, Lexington, KY USA

**Keywords:** Addiction prevention, Psychosocial competence, Positive youth development, Chinese adolescents, Primary school students, Program evaluation

## Abstract

Based on the evaluation findings of the B.E.S.T. Teen Program which aimed at promoting behavioral, emotional, social, and thinking competencies in primary school students, it is argued in this paper that promotion of psychosocial competence to prevent addiction in primary school students is a promising strategy. A total of 382 Primary 5 (Grade 5) and 297 Primary 6 (Grade 6) students from five primary schools in Hong Kong participated in the program. Different evaluation strategies were adopted to evaluate the program. First, objective outcome evaluation adopting a non-equivalent group pretest–posttest experimental-control group design was conducted to examine change in the students. Second, to gauge students’ perceptions of the program, subjective outcome evaluation was conducted. The evaluation findings basically converged to tentatively suggest that young adolescents benefited from participating in the program. Implications on the development, implementation, and evaluation of addiction prevention programs for teenagers are discussed.

## Background

In their “Handbook of Adolescent Health Risk Behavior”, DiClemente et al. (2013) painted a bleak picture on adolescent “risk behavior epidemic” (p. 3). They observed that the onset of risk behaviors such as alcohol, tobacco, and drug use was younger in the past decades. Moreover, an increasing proportion of high risk adolescents were found to be vulnerable to risk behaviors. Unfortunately, these emergent adolescent issues are not found only in Western societies. Shek and Leung (2013, 2014) reported alarming trends on problem behaviors including substance abuse and Internet addiction among Hong Kong youths. For example, the Narcotics Division of the Hong Kong Government found that 36.0 % of drug abusers claimed that they started drug use between the age of 12 and 15, and 3 % of students had reported doing so before the age of 12 (Narcotics Division 2013). In a recent survey of primary school students in Hong Kong (Lee 2015), it was found that children as young as 6 years old had admitted trying electronic cigarettes. As such, Lee suggested that preventive education should be conducted in the school context.

Mak et al. (2014) conducted a study on more than 5000 adolescents from six Asian regions including China, Hong Kong, Japan, South Korea, Malaysia, and the Philippines. They found that 68 % of Hong Kong students used the Internet at least once a day, which was the highest prevalence rate in all participating regions. The authors cautioned about the prevalent problematic Internet use and advised that early intervention is needed to “combat this new wave of addiction” (p. 726). It should be noted that Internet addiction is not currently recognized as a disorder by the psychiatric community and thus it is not included in the recently published Diagnostic and Statistical Manual of Mental Disorders V. However, it has been observed that many youngsters in the digital world exhibit symptoms and behaviors of addiction and suffer from a loss of sleep and poor academic performance (Wallace 2014). On the other hand, Boyd (2007) pointed out that not all Internet use among youths is dangerous. Appropriate and healthy use of Internet may help teenagers learn to form and enact a social identity and to be sensitive to social cues which are attempts of seeking access to the adult society.

Studies have also reported relatively widespread problem of gambling among young people. In Wong and So’s (2014) recent study on teenage gambling, they found that 63.5 % of Hong Kong secondary school students aged 12–19 reported that they had participated in offline gambling during the past year. The Home Affairs Bureau (2012) also reported that 1.8 % of secondary school and vocational training college students were classified as probable pathological gamblers, with 37.5 % of the respondents having taken part in gambling between ages 10 and 13, and 27.9 % began gambling before the age of 10.

### Psychosocial competence and addiction prevention

Masten and Coatsworth (1998) concluded that “prevention at its best represents both an effort to foster competence and to prevent problems” (p. 216). Proponents of positive youth development stressed that helping adolescents develop in different areas, including cognitive, social, moral, civic, vocational, cultural, and physical well-being, is an important strategy to reduce adolescent risk behavior (Pittman and Wright 1991). In addition, positive youth development approach adopts a strengths-based perspective which emphasizes strengths and assets of young people (i.e., capacities for positive development and protective factors) that keep them moving forward in a positive developmental path (Lerner and Galambos 1998).

Based on the above mentioned findings, Lam et al. (2011) argued that addiction prevention programs should focus on strengthening youths’ skills and abilities and help them develop their developmental assets. Indeed, studies have found that positive youth development promotes life satisfaction and reduces problem behavior among adolescents (Sun and Shek 2010, 2012). Specifically, Sun and Shek (2013) demonstrated that during middle-to-late adolescence, the effect of positive youth development on problem behavior is mediated by life satisfaction and concluded that “positive youth development is of paramount importance in enabling satisfaction with life and mitigating risk-taking behavior among early adolescents” (p. 471).

In fact, there is ample evidence to support the association between different positive youth development qualities (Catalano et al. 2002) and lowered adolescent addictive behaviors. Regarding intrapersonal competencies, lacking cognitive competence, such as poor decision making skills (Xiao et al. 2009), misperceptions in personal risks (Kropp and Halpern-Felsher 2004), illusory belief that one can control gambling events (Gupta and Derevensky 2000), and poor cognitive coping styles (e.g., Thompson 1991; Wills et al. 2001) contributed to addictive behaviors among youths. Emotional competence is also crucial to adolescent development as shown by the connection between poor emotional intelligence and addiction behaviors, such as gambling, gaming, internet use, tobacco, and alcohol use (e.g., Parker et al. 2008; Trinidad and Johnson 2002). For example, Chinese youths with Internet addiction were found to have weak emotional control and concentration skills (Breakthrough 2003).

Regarding interpersonal competencies, Caplan (2005) reported that individuals who lacked social skills were more likely to perceive online social interaction as favorable, which may lead to compulsive Internet use. Similarly, adolescents with gambling problems have been found to often experience difficulties in social and interpersonal interactions (Hardoon and Derevensky 2002). Finally, behavioral competence is essential for youths as it enables them to be assertive and express their feelings clearly, either verbally or non-verbally, and to act effectively in daily interactions with peers (Catalano et al. 2002). Studies have showed that peer pressure is often an impetus for adolescents to start experimenting with drugs or alcohol (Hansen and Graham 1991). Unwillingness to reject peers’ invitations and uneasiness of feelings of nonconformity among peer relationships can create pressure for young people to use drugs or drink alcohol. It is therefore argued that effective addiction prevention programs should adopt a positive youth development approach to nurture intrapersonal and interpersonal competencies of teenagers.

Catalano et al. (2002) summarized different youth development programs in the United States that had been evaluated and showed to yield significant positive impacts on children’s positive and problem behaviors. In the Asian context, Shek and Yu (2011) reviewed existing validated youth addiction prevention and positive youth development programs and identified 63 programs on adolescent prevention and positive development. They found that there are several limitations in existing programs, especially those in Hong Kong. Firstly, most programs were developed for adolescents (i.e., secondary school students) with very few programs targeting younger students in primary schools. As it is now known that some addictive behaviors such as gambling may have an early onset (Delfabbro et al. 2011), programs for primary school students are important. Secondly, many addiction prevention programs targeted single-problem behaviors. However, it is generally believed that risk behaviors in children and adolescents are interrelated especially in addictions. Thirdly, traditional prevention programs were found to be mostly problem-focused, and thus often had an over-pessimistic view on adolescents. In contrast to this, positive youth development programs were found to promote adolescent competence and had a more positive outlook on youth. Lastly, most existing prevention and positive youth development programs lacked rigorous evaluation. In an attempt to answer to these problems, the B.E.S.T. Teen Program was developed for Primary 5 and 6 (Grade 5 and 6) students in Hong Kong.

### Development of the B.E.S.T. Teen Program

First, a comprehensive review of existing addiction prevention and positive youth development programs targeting adolescents in both the Western and Eastern contexts was conducted. Members of the curriculum development team also reviewed literature regarding the determinants of effective addiction prevention programs (e.g., content, delivery mode) and closely examined existing programs especially in the Hong Kong context to ensure that the present program was distinctive, suitable for the local culture and for the target group of Hong Kong primary school students. Based on the findings of the review, it was decided that promotion of psychosocial competence of young people would be the best strategy to prevent addiction.

The multi-addiction prevention program adopts a positive youth development approach and aims to introduce knowledge of addiction behaviors to targeted students and promoting students’ intrapersonal (e.g., cognitive and emotional) and interpersonal (e.g., social and behavioral) competencies, which serve as protective factors against risk behaviors. The B.E.S.T. Teen Program consists of five components related to positive youth development: (1) equip students with knowledge on the nature of addiction and remove their misconceptions about addiction; (2) promote cognitive competence; (3) promote emotional competence; (4) promote social competence; and (5) promote behavioral competence. Because of differences in students’ cognitive development, there are different curriculum materials for Primary 5 and 6 students. The curriculum is composed of five parts, which are then further divided into two units. Each unit takes 30 min, totaling to 5 h. The curriculum, teaching goals, and intended learning outcomes for Primary 5 and 6 students are presented in Tables 1 and 2.

**Table 1:** Primary 5 curriculum, teaching aims, and learning outcomes

Construct	Unit	Teaching aims	Learning outcomes After taking this unit, students will be able:
Nature of addiction	NA5.1	To facilitate students to acquire knowledge about different types of addiction and common signs of addiction	To understand common characteristics of substance abuse and behavioral addiction To identify drug tolerance and withdrawal as common signs of addiction
	NA5.2	To enhance students’ awareness of short term and long term consequences of addiction	To identify short and long term negative physiological, psychological, and social impacts of addiction
Cognitive competence	CC5.1	To clarify the misconceptions of addiction	To identify facts and misconceptions of addiction
	CC5.2	To cultivate effective decision making	To understand factors affecting decision making To differentiate effective from ineffective decision making
Emotional competence	EC5.1	To introduce different emotions	To understand how different emotions are experienced To distinguish different emotional states (i.e., positive and negative emotions)
	EC5.2	To familiarize students with healthy ways to foster positive emotions	To use effective methods to experience positive emotions
Social competence	SC5.1	To introduce different types of peers and possible peer influences	To differentiate between good friends and bad friends
	SC5.2	To introduce basic interpersonal skills in social interaction	To understand the skills on assertiveness
Behavioral competence	BC5.1	To explain peer pressure and its relation to group conformity	To understand situations involving peer pressure
	BC5.2	To learn how to stay away from addiction	To be able to say “no” to addictive agents

**Table 2:** Primary 6 curriculum, teaching aims, and learning outcomes

Construct	Unit	Teaching aims	Learning outcomes After taking this unit, students will be able:
Nature of addiction	NA6.1	To facilitate students to acquire knowledge on different types of addiction and understand the connection between addiction and the body	To understand common characteristics of substance abuse and behavioral addiction To identify the role the brain plays in addiction
	NA6.2	To enhance students’ awareness of short term and long term consequences of addiction	To identify short and long term negative physiological, psychological, and social impacts of addiction
Cognitive competence	CC6.1	To clarify the misconceptions of addiction	To identify misconceptions of addiction To understand factors leading to addictive behaviors
	CC6.2	To cultivate effective decision making and positive coping strategies	To differentiate effective from ineffective decisions To learn healthy cognitive coping strategies, such as positive thinking styles
Emotional competence	EC6.1	To introduce different emotions	To identify how different emotions are experienced To understand the antecedents and consequences of emotions
	EC6.2	To familiarize students with healthy ways to foster positive emotions	To use effective methods to experience positive emotions To enhance emotional self-efficacy
Social competence	SC6.1	To introduce different types of peers and possible peer influences	To identify undesirable peer influence To select friends in a careful manner
	SC6.2	To introduce basic interpersonal skills in social interaction	To express one’s thoughts and feelings in social situations To understand interpersonal conflict and its consequences
Behavioral competence	BC6.1	To introduce peer pressure and its relation to group conformity	To identify situations involving peer pressure To explore ways to deal with conformity
	BC6.2	To learn how to stay away from addiction	To understand impulse control To explore ways to control impulsive addictive behavior

When the curriculum was developed, two primary school teachers and two social workers were invited to attend a focus group where they shared comments on the lesson plans of the curriculum. On the whole, the teachers and social workers viewed the curriculum positively. They saw the content as relevant, the curriculum appropriate for the students and the activities as engaging. They also provided feedback on how to improve the curriculum (e.g., a need to provide more detailed definition of certain terms) and informed the researchers on activities that students usually find engaging. The curriculum development team then further revised the curriculum based on these comments.

### Program implementation

The program was implemented for Primary 5 and 6 students in the randomly selected schools. The duration of implementation varied across schools ranging from two-day intensive implementation to one month gradual implementation. Implementation team of the B.E.S.T. Teen Program included registered social workers, psychologists with Bachelor’s and postgraduate degree and researchers who had ample training and experience in teaching and implementation of positive youth development and addiction prevention programs. The programs were implemented during students’ life education lessons or unstructured periods. A teacher from the school introduced to the students the implementer who was then responsible for leading the lesson. Table 3 details the background information of the participating students.

**Table 3:** Demographics of program participants and respondents

	Primary 5			Primary 6			Total	
	Experimental group	Control group	Total	Experimental group	Control group	Total	Experimental group	Control group
Program participants	382	234	778	297	333	630	679	567
Male	110	85	195	109	133	242	219	218
Female	112	79	191	102	112	214	214	191
Mean age (SD)	11.16 (.54)	11.38 (.65)	11.25 (.60)	12.37 (.68)	12.40 (.64)	12.39 (.66)	11.75 (.86)	11.99 (.81)
Pretest questionnaires collected^a^	222	164	386	211	245	456	433	409
Posttest questionnaires collected^a^	215	164	379	210	244	454	425	408
Subjective outcome evaluation	373	–	–	271	–	–	644	–

^a^Objective outcome evaluation

## Methods

### Participants and procedures

Recruitment of participants to the B.E.S.T. Teen Program was conducted in the 2013–2014 school year. Over 50 local primary schools in Hong Kong were contacted including government schools, as well as government aided schools operated by the Tung Wah Group of Hospitals. A total of 37 primary schools in Hong Kong expressed their interest in joining the Program. Among the interested parties, five primary schools were randomly selected to participate in the program as the experimental group and five primary schools with similar background (district, school banding, and student composition) as the experimental schools were selected as the control group. Selected primary schools were all from districts in the New Territories. The program was implemented in the 2013–2014 school year. A total of 382 Primary 5 and 297 Primary 6 students participated in the program (see Table 3). Letters were sent to parents via respective schools to obtain their consent for students to take part in the evaluation study.

When collecting the data, the research staff explained the purpose of the study and re-assured the students of the confidentiality of their answers. Participants responded to different scales in a self-administration format. Sufficient time was provided for the participants to complete the questionnaires. All questionnaires were administered and conducted by trained research staff. Upon the completion of the questionnaires, students were clearly debriefed and the purpose of the evaluation studies was further highlighted. For the control group (i.e., who did not participate in the B.E.S.T. Teen Program), a 1 h workshop was delivered to the students after the data collection had been completed. The workshop offered basic knowledge of addiction, common misconceptions about addiction, and a brief introduction of the intrapersonal and interpersonal competencies.

#### Evaluation methods

Different methods were used to examine the effectiveness of the program. At the first stage, in order to examine whether students changed after taking part in the program, analyses of the data collected from the non-equivalent group pretest–posttest experimental-control group design was carried out. For the experimental group, pretest questionnaires were administered to students before the first session of the program, and posttest questionnaires were handed out after the completion of the final session. For the control group, questionnaire surveys were conducted twice at the same time with the experimental group. Second, to find out students’ perceptions of the program, a subjective outcome evaluation form was given to participants after completing the final session. Qualitative evaluation methods were also used in the study. However, because of space limitation, related qualitative findings will be reported elsewhere.

### Instruments

#### Objective outcome evaluation

Based on existing literature, validated questionnaires (e.g., Young 1999; Shek et al. 2008; Shek and Lam 2008), expert views, and intended learning outcomes of the Program curriculum, an objective outcome evaluation questionnaire was developed. The questionnaire consisted of 57 items assessing participants in five domains of addiction:

- *Part A: Addictive Behavior (eight items)* Participants were invited to indicate their frequency of showing different types of addictive behavior in the past month, including smoking, drinking alcohol, using cough mixture without medical condition, inhaling the fumes from organic solvent, using Ketamine, using Heroin, gambling, and uncontrollable Internet use. Respondents rated the frequency of these behaviors in the past month on a 6-point Likert scale (0 = *never*; 1 = *one time*; 2 = *two times*; 3 = *three times*; 4 = *one time per week*; 5 = *several times per week*; 6 = *everyday*). It should be noted that while there are controversies regarding the definition and classification criteria of Internet addiction, it was defined as one’s uncontrollable use of the Internet or computer games even the use has negatively affect one’s life (Davis 2001; Young 1999). The item used to measure participants’ Internet addictive behavior reads as “feeling hard to control time spending on the Internet or computer games even it has negatively affected my life (e.g., health, study, and family relationship)”. Although the measures were internally consistent in adolescent samples (Shek and Yu 2012), reliability was not high in this study. Cronbach’s α ranged from .24 to .31 at pretest and posttest for the experimental group and the control group. Therefore, data analyses were based on individual item scores instead of the scale score.
- *Part B: Behavioral Intention (eight items)* Respondents were asked to assess on a 4-point Likert scale (1 = *never*; 2 = *not likely*; 3 = *likely*; 4 = *definitely*) the likelihood that they might engage in the above eight addictive behaviors in the next 2 years. Example items are “would you smoke cigarettes in the coming 2 years?” and “would you spend most of your time on the Internet or computer games even it will negatively affect your life?” Cronbach’s alpha for the scale ranged from .36 to .37 for both the experimental group and the control group at pretest and posttest. Similar to Part A, individual item scores instead of the scale scores were used for data analysis.
- *Part C: Psychosocial Competencies (12 items)* Participants were invited to indicate their agreement on items describing skills relevant to different positive youth development competencies (e.g., cognitive, emotional, social, and behavioral competence). For example, one question assessing the behavioral competence was “I will consider all choices before I make decisions”. Respondents rated their perceptions on a 6-point Likert scale ranging from 1 = *strongly disagree* to 6 = *strongly agree*. Cronbach’s α of pretest was = .68 and for posttest it was .69.
- *Part D: Knowledge about Addiction (16 items)* Students were given 16 claims to assess their knowledge on addiction. Response options were true, false, and unsure. Some examples included: “There are many different types of addiction” and “There are many factors that may lead to addiction”. While students in the control group did not participate in the Program, they were provided with the correct answers after the posttest.
- *Part E: Beliefs about Addiction (13 items)* Students were asked how much they agreed with statements describing different beliefs about addiction. Example statements included, “It is fine for children of my age group to smoke” (reverse coding); and “People shall control their time spent on the Internet or computer games”. Respondents rated the items on a 6-point Likert scale ranging from 1 = *strongly disagree* to 6 = *strongly agree*. Cronbach’s α for pretest was .73 and for posttest it was .76.

#### Subjective outcome evaluation

The subjective outcome evaluation comprised of 38 items evaluating participants’ perceptions regarding the following three attributes:

- *Part A: Program Attributes* (ten items; α = .96) Program attributes included program objectives, content, student interaction, classroom atmosphere, and encouragement received during lessons.
- *Part B: Implementer Attributes* (ten items; α = .97) Implementer attributes included implementers’ preparedness, professional attitude, knowledge, and interaction with participants.
- *Part C: Program Effectiveness* (12 items; α = .96) Items evaluating program effectiveness included promotion of different psychosocial competencies, overall personal development, and achievement of the intended learning outcomes.

For Program and Implementer Attributes, participants were invited to rate on a 6-point Likert Scale the extent to which they agreed with the items (ranging from 1 = *strongly disagree* to 6 = *strongly agree*). For Program Effectiveness, participants indicated the extent to which the program had helped to enhance their skills and competencies (ranging from 1 = *no help* to 5 = *great help*).

In addition, two items were included in the subjective outcome evaluation form to examine whether (1) students would recommend the program to their friends with similar backgrounds/needs; and whether (2) they would participate in similar programs in the future. Moreover, students’ overall satisfaction towards the program was measured using a single item with ratings ranging from 1 (*highly dissatisfied)* to 6 (*highly satisfied)*.

The subjective outcome evaluation scale was previously validated in an adolescent population and shown to be valid and reliable (Shek and Yu 2014).

### Data analyses strategy

#### Objective outcome evaluation

The outcome measures were divided into five categories: (1) addictive behaviors; (2) behavioral intentions; (3) psychosocial competencies; (4) knowledge about addiction, and (5) beliefs about addiction.

First, experimental group and control group were compared at pretest in terms of the five categories. For “addictive behavior”, percentages of students who reported “never” showing the behavior were compared between the two groups based on Chi square tests. Independent samples t-tests were conducted to examine the differences in item scores of “behavioral intention” and scale scores of “psychosocial competencies”, “knowledge about addiction”, and “beliefs about addiction” between experimental and control groups.

Second, a series of regression analyses were performed to evaluate whether participation in the program predicted students’ posttest objective outcome indicator scores. A total of eight logistic regression models and 11 multiple regression models were estimated. For the eight logistic models, each “addictive behavior” (0 = *never*; 1 = *at least once in the past month*) served as the dependent variable in one model. For the multiple regression models, the mean scores of each “behavioral intention” item (totally eight), the “knowledge about addiction” scale, “psychosocial competencies” scale, and “beliefs about addiction” scale served as the dependent variables. For independent variables, gender (0 = *male*; 1 = *female*) and age were entered at the first block; students’ pretest score was entered at the second block, and group (0 = *control*; 1 = *experimental*) was entered at the third block.

#### Subjective outcome evaluation

For the subjective outcome evaluation, descriptive profiles based on frequencies and proportions were examined using descriptive statistics. Findings from the close-ended items are reported in this paper in order to provide the readers an overall profile of the data.

## Results and discussion

The present study examined the effectiveness of the B.E.S.T. Teen Program, a multi-addiction prevention program targeting Primary 5 and 6 students in Hong Kong. To investigate whether students who participated in the program had changed, objective outcome evaluation was conducted.

Tables 4 and 5 show the results of pretest comparison between the experimental group and control group in terms of the five categories. For addictive behaviors, the proportions of students who reported never “drink alcohol”, “gamble”, and have “uncontrollable Internet use” in the experimental group were significantly higher than the proportions in control group. Similarly, students of experimental group showed less intention to “drink”, “gamble”, and “use Internet excessively” in the coming 2 years than students of control group. Meanwhile, experimental group scored higher on their psychosocial skills, knowledge and correct beliefs about addiction than did control group. These results suggest that students in the experimental group may have a relatively better development than did the control group in the five areas measured before the Program was implemented. Hence, there was a need to control the pretest scores for the related measures when we compared the two groups.

**Table 4:** Pretest comparison in addictive behaviors between experimental and control groups

	Control group							Experimental group							*χ* ^2^ statistics
	0	1	2	3	4	5	6	0	1	2	3	4	5	6	
Smoking	383 (96.7 %)	9 (2.3 %)	1 (0.3 %)	3 (0.8 %)	0 (0 %)	0 (0 %)	0 (0 %)	402 (98.3 %)	6 (1.5 %)	1 (0.2 %)	0 (0 %)	0 (0 %)	0 (0 %)	0 (0 %)	2.05
Drinking	274 (69.2 %)	57 (14.4 %)	24 (6.1 %)	33 (8.3 %)	4 (1.0 %)	4 (1.0 %)	0 (0 %)	318 (77.8 %)	44 (10.8 %)	24 (5.9 %)	16 (3.9 %)	6 (1.5 %)	1 (0.2 %)	0 (0 %)	7.57**
Cough mixture misuse	376 (94.9 %)	8 (2.0 %)	6 (1.5 %)	4 (1.0 %)	0 (0 %)	2 (0.5 %)	0 (0 %)	397 (97.1 %)	3 (0.7 %)	5 (1.2 %)	2 (0.5 %)	1 (0.2 %)	1 (0.2 %)	0 (0 %)	2.36
Inhaling organic solvent	393 (99.2 %)	3 (0.8 %)	0 (0 %)	0 (0 %)	0 (0 %)	0 (0 %)	0 (0 %)	400 (97.8 %)	4 (1.0 %)	1 (0.2 %)	4 (1.0 %)	0 (0 %)	0 (0 %)	0 (0 %)	2.85
Ketamine use	396 (100 %)	0 (0 %)	0 (0 %)	0 (0 %)	0 (0 %)	0 (0 %)	0 (0 %)	409 (100 %)	0 (0 %)	0 (0 %)	0 (0 %)	0 (0 %)	0 (0 %)	0 (0 %)	NA
Heroin use	396 (100 %)	0 (0 %)	0 (0 %)	0 (0 %)	0 (0 %)	0 (0 %)	0 (0 %)	409 (100 %)	0 (0 %)	0 (0 %)	0 (0 %)	0 (0 %)	0 (0 %)	0 (0 %)	NA
Gambling	325 (82.1 %)	37 (9.3 %)	16 (4.0 %)	9 (2.3 %)	7 (1.8 %)	1 (0.3 %)	1 (0.3 %)	373 (91.2 %)	15 (3.7 %)	5 (1.2 %)	11 (2.7 %)	2 (0.5 %)	3 (0.7 %)	0 (0 %)	14.54***
Uncontrollable Internet use	260 (65.7 %)	66 (16.7 %)	16 (4.0 %)	10 (2.5 %)	7 (1.8 %)	20 (5.1 %)	17 (4.3 %)	315 (77.0 %)	53 (13.0 %)	10 (2.4 %)	8 (2.0 %)	6 (1.5 %)	3 (0.7 %)	14 (3.4 %)	12.72***

0 = never; 1 = one time; 2 = two times; 3 = three times; 4 = one time per week; 5 = several times per week; 6 = everyday

*χ* ^2^ comparison was based on the proportions of students who scored “0” (“never”) in the two groups

** *p* < .01; *** *p* < .001

**Table 5:** Pretest comparison between experimental and control groups in behavioral intention, psychosocial skills, knowledge, and beliefs

	Experimental group	Control group	t
	Mean (SD)	Mean (SD)	
Behavioral intention			
Smoking	1.05 (0.30)	1.07 (0.33)	−0.87
Drinking	1.37 (0.70)	1.54 (0.86)	−3.16**
Cough mixture misuse	1.06 (0.33)	1.06 (0.33)	0.19
Inhaling organic solvent	1.04 (0.25)	1.03 (0.25)	0.22
Ketamine use	1.02 (0.19)	1.01 (0.16)	0.57
Heroin use	1.03 (0.25)	1.02 (0.22)	0.56
Gambling	1.13 (0.43)	1.20 (0.53)	−2.27*
Uncontrollable Internet use	1.66 (0.85)	1.91 (0.94)	−4.08***
Skill acquisition	5.00 (0.65)	4.85 (0.68)	3.06**
Knowledge	12.12 (2.85)	11.33 (3.30)	3.62***
Beliefs	5.26 (0.65)	5.13 (0.75)	2.63**

Values in the tables are Mean (standard deviation) of students’ scores

* *p* < .05; ** *p* < .01; *** *p* < .001

Results of logistic regression analyses showed that group significantly predicted the occurrence of uncontrollable Internet use in the participants after controlling for the effects of gender, age, and pretest scores (*B* = −0.61, *S.E.* = 0.19, *OR* = 0.55, *p* < .002). In other words, students who participated in the program were 45 % less likely to display uncontrollable Internet use than did students who didn’t participate in the program. No significant effect of group was found on other addictive behavior indicators. For multiple regression analyses, after controlling for the effects of gender, age, and pretest scores, group significantly predicted participants’ intention to use Internet excessively in the coming 2 years (*β* = −.07, *p* = .02) and predicted smoking intention with borderline significance (*β* = −.06, *p* = .07). Experimental group students reported a lower intention to use Internet excessively than did control group students, and there was a tendency that students participating in the program had a lower intention to smoke in the future than control group. For other objective outcome indicators, no significant group effect was identified. In sum, objective outcome evaluation provides partial support for the effectiveness of the program.

There are several possible explanations for the mixed findings obtained. First, experimental group performed better than control group at pretest in all five categories of the objective outcomes. Hence, there might be more room for the control group to improve, especially in a relatively short period of time. In other words, “regression to the mean” may explain the findings. Second, the occurrence rate of some addictive behaviors measured in the present study was quite low in primary school students, such as the use of Ketamine and heroin. That is, floor effect may explain the findings. Third, developmental transitions such as puberty and increasing independence, and limitations in brain development during adolescence have been associated with teenagers’ increased tendency of risk taking (Arnett 1992). The prevalence of risk behaviors (e.g., alcohol use) in early adolescence (10–15 years) has been found to be positively related with age (MacArthur et al. 2012). As the present program was only implemented over the span of one semester, there may be a limitation in the scope of what can be achieved during such a short duration. Obviously, further studies should be done to clarify these issues.

Findings from the subjective outcome evaluation are presented in Tables 6 and 7. Regarding Primary 5 students’ responses, respondents generally held highly positive views toward the curriculum content, where majority of the participants rated the items positively (ranging from 89.7 to 94.3 %). Students perceived the objectives to be clear (94.3 %) with a good curriculum design (92.7 %). Students were also highly satisfied with the program implementers as over 90 % of them rated the items assessing implementers to be positive. Students reported that the implementers demonstrated good teaching skills (96.5 %) and had much interaction with students (93.8 %). Most importantly, students believed that the program had helped them gain more knowledge on addiction (95.4 %), enhanced their ability to make analysis (96.8 %) and good decisions (96.5 %), helped them manage (95.9 %) and be aware of (93.8 %) their emotions, and helped them develop healthy behavior (96.7 %) and resist negative influence (96.2 %).

**Table 6:** Summary of students’ perceptions toward the curriculum content and implementers

Item	Primary 5			Primary 6		
	Mean (SD)	Positive responses (4–6)		Mean (SD)	Positive responses (4–6)	
		N	%		N	%
*Curriculum content*						
1. The objectives of the curriculum are very clear	5.22 (0.97)	350	94.3	5.16 (1.09)	251	92.6
2. The content design of the curriculum is very good	5.03 (1.00)	344	92.7	4.96 (1.17)	243	89.7
3. The activities were carefully arranged	5.10 (0.98)	348	93.8	5.04 (1.18)	248	91.9
4. The classroom atmosphere was very pleasant	5.06 (1.14)	338	91.6	4.94 (1.19)	244	90.0
5. There was much peer interaction amongst the students	5.02 (1.15)	331	89.7	5.04 (1.19)	243	91.0
6. I participated in the class activities actively (including discussions, sharing, games, etc.)	5.13 (1.06)	347	93.5	5.06 (1.21)	243	90.3
7. I was encouraged to do my best	4.88 (1.18)	332	89.7	4.91 (1.22)	246	91.1
8. The learning experience enhanced my interests towards the program	4.96 (1.17)	334	90.5	4.89 (1.21)	240	88.6
9. All in all, I have a very positive evaluation on the program	4.95 (1.16)	334	90.3	4.93 (1.18)	248	91.5
10. On the whole, I like this program very much	5.10 (1.11)	335	91.0	4.98 (1.18)	246	91.1
*Implementers*						
1. The implementer(s) had a good mastery of the program	5.31 (0.94)	354	95.4	5.25 (1.10)	256	94.5
2. The implementer(s) was (were) well prepared for the lessons	5.37 (0.95)	354	95.4	5.30 (1.07)	257	95.2
3. The teaching skills of the implementer(s) were good	5.32 (0.93)	356	96.5	5.25 (1.10)	253	94.1
4. The implementer(s) showed good professional attitudes	5.44 (0.90)	361	97.3	5.31 (1.10)	253	94.8
5. The implementer(s) was (were) very involved	5.42 (0.91)	359	96.8	5.32 (1.08)	257	94.8
6. The implementer(s) encouraged students to participate in the activities	5.36 (0.91)	356	96.7	5.29 (1.08)	253	93.7
7. The implementer(s) cared for the students	5.33 (0.96)	355	96.2	5.27 (1.09)	256	94.8
8. The implementer(s) was (were) ready to offer help to students when needed	5.39 (0.95)	355	95.9	5.35 (1.07)	258	95.2
9. The implementer(s) had much interaction with the students	5.29 (1.03)	346	93.8	5.18 (1.12)	254	93.7
10. Overall speaking, I have a very positive evaluation of the implementer(s)	5.37 (0.96)	357	96.5	5.34 (1.08)	258	95.2

All items are rated on a 6-point Likert scale with 1 = strongly disagree, 2 = disagree, 3 = slightly disagree, 4 = slightly agree, 5 = agree, 6 = strongly agree. Only respondents with positive responses (Options 4–6) are shown in the table

**Table 7:** Summary of students’ perceptions of the program effectiveness

Item	Primary 5			Primary 6		
	Mean (SD)	Positive responses (4–5)		Mean (SD)	Positive responses (4–5)	
		N	%		N	%
*Program effectiveness*						
1. Enhanced my knowledge on the nature of addiction	4.24 (0.86)	354	95.4	4.24 (0.94)	227	84.4
2. Heightened my awareness of addiction	4.38 (0.84)	354	95.4	4.30 (0.91)	231	86.2
3. Enhanced my competence in differentiating right from wrong	4.33 (0.88)	356	96.5	4.33 (0.86)	231	86.2
4. Strengthened my ability to make wise decisions	4.33 (0.86)	361	97.3	4.34 (0.89)	228	85.1
5. Enhanced my analytical skills	4.26 (0.90)	359	96.8	4.31 (0.88)	229	85.8
6. Helped me to develop healthy behavioral habits	4.37 (0.89)	356	96.7	4.31 (0.90)	229	85.8
7. Improved my ability to resist negative influence	4.31 (0.93)	355	96.2	4.33 (0.90)	229	85.1
8. Strengthened my ability to manage emotions	4.18 (0.93)	355	95.9	4.20 (0.98)	210	78.1
9. Helped me to become aware of my emotions	4.23 (0.94)	346	93.8	4.24 (0.96)	216	80.6
10. Nurtured my ability to interact with others	4.22 (0.96)	357	96.5	4.20 (0.96)	214	79.9
11. Encouraged me to strengthen my bonds with teachers, students, and my family	4.10 (1.02)	353	95.8	4.13 (1.03)	213	79.2
12. Promoted my overall development	4.28 (0.93)	353	95.7	4.24 (0.99)	217	81.3

All items are rated on a 5-point Likert scale with 1 = not at all helpful, 2 = not very helpful, 3 = somewhat helpful, 4 = helpful, 5 = very helpful. Only respondents with positive responses (Options 4–5) are shown in the table

Similarly, Primary 6 students were also highly satisfied with different aspects of the program as evidenced by the high percentages of positive ratings in terms of the curriculum content (88.6–92.6 %), program implementers (over 90 % across all items), and program effectiveness (78.1–86.2 %). Students reported that their overall personal development was improved (81.3 %). The findings from the subjective outcome evaluation from both Primary 5 and 6 students showed that the program was well-received and that the intended learning outcomes of the curriculum were achieved. The findings from the subjective outcome evaluation illustrate the important roles of curriculum content and implementers in successful implementation of prevention programs.

The experiences and findings that are gained through implementing and evaluating the B.E.S.T. Teen program have led us to make several recommendations regarding the development and implementation of future youth addiction prevention programs. First, it is suggested that a positive youth development approach should be adopted in addiction prevention. Recently, researchers and practitioners agreed that adolescent problem behaviors including addictive behaviors share common antecedents and that prevention science should adopt a broader focus to address both risk and protective factors (Catalano et al. 2002). Shek (2007) argued that one of the factors that contribute to adolescent substance abuse in Hong Kong is the lack of psychosocial competencies and coping skills. To tackle the problem, it is recommended that systematic holistic positive youth development programs should be implemented, especially for young adolescents. In fact, a large-scale curricula-based positive youth development program in Hong Kong entitled Project P.A.T.H.S. (Positive Adolescent Training through Holistic Social Programmes) was developed and successfully implemented. Shek and Yu (2012) reported evidence which demonstrated that the Project P.A.T.H.S. yielded long-term effect in preventing adolescent problem behavior including delinquency, use of drugs, and lowering intentions in participating in risk behaviors, which underscores the effectiveness and importance of adopting a positive youth development approach to the prevention of adolescent risk behaviors and promotion of positive development.

Second, addiction prevention programs should begin in primary school and target young adolescents. Existing addiction prevention programs for youths mainly target secondary school students. In Hong Kong, there is also a severe lack of systematic holistic positive youth development programs especially targeted at young adolescents (Shek 2007). Therefore, addiction prevention and positive youth development programs should widen their target scope to include not only secondary school students, but also primary school students.

Third, findings based on different evaluation strategies demonstrated that interactive delivery methods (e.g., role-plays, video watching, reflection exercises, and drawing) were well-received by students and helped to benefit prevention efforts. Lastly, in a review of school-based prevention programs, Durlak and Wells (1997) noted that few evaluation studies adopted a longitudinal design to investigate the sustainability of the impact. Therefore, it is suggested that future evaluation of youth addiction prevention programs should include follow-up assessments to investigate whether the impacts of the program are long lasting.

While the findings from both objective and subjective evaluation provided support to the effectiveness of the B.E.S.T. Teen program, the present study has several limitations. First, due to limited resources, timetabling of different schools and other reasons, not all students who took part in the program joined the evaluation (e.g., some students had to attend inter-school competitions). It would be desirable to recruit a larger sample in the different areas of evaluation. Second, addictive behaviors were measured by single items. Psychometrically, sound measures must be adopted in future to provide a more accurate estimation about teenagers’ addictive behaviors. For example, it would be helpful to use Young’s Internet Addiction Test (Young 1999) to measure Internet addictive behaviors. Third, the validity of information obtained via self-report methods has been questioned. However, taking into consideration ethical and practical concerns and sensitivity of the topic, especially for primary school students, self-report measures are not unreasonable. Fourth, the research team is aware of that the participants of the study are primary school students who may have difficulty in comprehending certain items. Therefore, trained research staff was present in all data collection sessions and welcomed students to raise questions where clarifications were needed. In fact, client satisfaction questionnaires have been successfully administered to primary school students in the past (Yun and Lyu 2012).

Finally, it is noteworthy that the Cronbach alphas for adolescent past addictive behaviors and intention to engage in addictive behavior subscales suggested low internal reliability. Interestingly, such scales have been found to be valid and reliable in previous studies on early adolescent population in Hong Kong (Shek and Yu 2012). There might be few reasons to explain the different results in the present study. First, reliability analyses revealed low variance across items of both past experience and intention to engage in addictive behaviors. Particularly, all participants responded that they had no experience or intention in taking substances such as heroine and ketamine. Furthermore, the items on the scales represent distinct behaviors under the same conceptual umbrella of addiction which may not necessarily tap into any specific underlying construct. Although they are addictive behaviors, they may not occur at the same time. Thus, internal consistency may not be an appropriate measure of the reliability (Goodman et al. 2003). In addition, only eight items were included in each of the subscales. In light of the above, the data collected in the present study should be viewed as preliminary. In order to better understand adolescent addictive behaviors and intention, future studies should include more items assessing specific addictive behaviors and intentions (e.g., sports betting, buying the lottery, horse betting). In spite of the above limitations, the data collected using different strategies provided some support for the effectiveness of the B.E.S.T. Teen Program.

## Conclusion

As few evaluation studies have been conducted on multi-addiction prevention programs targeted at primary school students, especially in Hong Kong, the B.E.S.T. Teen Program and the evaluation study were developed. The program and the related evaluation study can be regarded as pioneer but the evaluation findings should be regarded as preliminary. Based on the objective outcome and subjective outcome evaluation findings, the present study provides some initial support for the effectiveness of the program, although it is noteworthy that there are several limitations of the study. Obviously, recommendations put forward in the present paper may serve to inform program developers and practitioners on how to design and implement effective prevention programs. Furthermore, more studies should be conducted to replicate and extend the findings arising from the present study.
